# Competitive Adsorption of Metals onto Magnetic Graphene Oxide: Comparison with Other Carbonaceous Adsorbents

**DOI:** 10.1155/2015/836287

**Published:** 2015-03-12

**Authors:** Jin Hur, Jaewon Shin, Jeseung Yoo, Young-Soo Seo

**Affiliations:** ^1^Department of Environment & Energy, Sejong University, Seoul 143-747, Republic of Korea; ^2^Department of Nano Science & Technology, Sejong University, Seoul 143-747, Republic of Korea

## Abstract

Competitive adsorption isotherms of Cu(II), Pb(II), and Cd(II) were examined on a magnetic graphene oxide (GO), multiwalled carbon nanotubes (MWCNTs), and powered activated carbon (PAC). A series of analyses confirmed the successful synthesis of the magnetic GO based on a simple ultrasonification method. Irrespective of the adsorbents, the adsorption was highly dependent on pH, and the adsorption was well described by the Langmuir isotherm model. The maximum adsorption capacities of the adsorbents were generally higher in the order of Pb(II) > Cu(II) > Cd(II), which is the same as the degree of the electronegativity and the hydrated radius of the metals, suggesting that the metal adsorption may be governed by an ion exchange between positively charged metals and negatively charged surfaces, as well as diffusion of metals into the surface layer. The adsorption of each metal was mostly lower for multi- versus single-metal systems. The antagonistic effects were influenced by solution pH as well as the type of metals, and they were higher in the order of the magnetic GO > MWCNT > PAC. Dissolved HS played a greater role than HS adsorbed onto the adsorbents, competing with the adsorption sites for metal complexation.

## 1. Introduction

Due to growing industrialization and urbanization, heavy metals are increasingly introduced into aquatic environments via various pathways. Heavy metals are not bioavailable and tend to accumulate in living tissues, thereby threatening human health and aquatic ecosystems. A variety of the treatment technologies have been proposed and used to eliminate heavy metals from polluted water. Such technologies are mostly based on adsorption, chemical precipitation, ion exchange, membrane filtration, reverse osmosis, and electrolysis [1]. Among those, adsorption can be ranked as one of the most preferred methods for treating heavy metals because of its high efficiency in the cost and the operation. The choice of adsorbents is the key to successful application for the adsorption-based treatments. Activated carbon, inorganic minerals, and bioadsorbents have been studied as popularly used adsorbents [1]. Recently, carbon nanotubes (CNTs) and graphene materials emerged as promising adsorbents for heavy metal removal because of their high specific surface areas and chemically stable structures [2, 3].

Graphene oxide (GO), prepared by oxidation of natural graphite, is a two-dimensional nanomaterial bearing several functional groups such as hydroxyl, epoxide, carbonyl, and carboxyl groups on its basal planes and sheet edges [4]. The large surface area and the abundance of the oxygen-containing functional groups make GO highly attractive for the removal of heavy metals from polluted water. However, good dispersive property of GO in aqueous phases has been regarded as an obstacle for separating and retrieving the adsorbent for reuse after treating heavy metals. Recently, combining graphene with the magnetic materials such as Fe_3_O_4_ was suggested to overcome this limitation [3, 5–12]. Furthermore, Fe_3_O_4_ is known to have low toxicity and good biocompatibility, which is advantageous for water treatment in practice [13].

In many cases, the removal efficiency of adsorbents is evaluated based on the adsorption capacity for a single-metal system, which contrasts with the common observation of the industrial wastewater or other polluted water sources containing a mixture of several heavy metals. Therefore, it is desirable for the full applications in practice to examine the adsorption behavior of the individual metals under multimetal systems, in which more than two types of metals exist together and they might compete with each other for the same adsorption sites. A comparison between single- and multimetal systems for the adsorption performance can ultimately provide a guideline for choosing the most feasible adsorbents for the water contaminated with a mixture of heavy metals, as well as for seeking the optimum operating conditions.

The adsorption of metals onto activated carbon (AC) and CNTs, which have been frequently studied for the removal of heavy metals, is well documented [2, 14]. It was reported that acidic functional groups distributed on the adsorbent's surfaces play critical roles in binding metals through ion exchange, electrostatic attraction, and sorption-precipitation processes [2]. Magnetic GO is recently highlighted as an effective adsorbent for treating heavy metals, while further investigation is required to warrant the widespread use in practice [9]. There are only limited adsorption studies using magnetic GO for multimetal systems [15]. A few studies have attempted to compare several adsorbents including GO for their metal adsorptive capabilities [10, 15]. However, they simply adopted the results from other studies to highlight the advantages of their own materials without a rigorous comparison based on the experiments. It is noteworthy that the adsorption performances of different adsorbents should be compared under the same experimental conditions in order to provide the exact information on the metal selectivity and adsorption affinities. The objectives of this study are (1) to compare the adsorption behaviors of Cu(II), Pb(II), and Cd(II) for three different carbonaceous materials (i.e., AC, CNT, and magnetic GO) under the identical experimental condition and (2) to explore the competitive adsorption behaviors of the metals on each adsorbent by comparing the adsorption behaviors between single- and multimetal systems.

## 2. Material and Methods

### 2.1. Preparation of Graphite Oxide

Graphite oxide was prepared based on a modification of the Hummers method using graphite flakes [16]. For pretreatment, 12 g of graphite flakes (Aldrich) was added into 50 mL of a preheated sulfuric acid (H_2_SO_4_) solution containing 10 g each of potassium persulfate (K_2_S_2_O_8_, Sigma-Aldrich) and phosphorus pentoxide (P_2_O_5_, Sigma-Aldrich). They were mixed at 80°C for 5 hours. The mixture was left at room temperature overnight after 2 L of Milli-Q water was added. The pretreatment was completed by filtering the mixture through 5 *μ*m pore-sized filters (polyester fiber). For further treatment, 2 g of the purified graphite, 2 g of NaNO_3_, and 46 mL of sulfuric acid were mixed together in a flask in an ice bath below 10°C. 6 g of KMnO_4_ (Sigma-Aldrich) was added, slowly mixed, and left for reaction in a water bath at 35°C for 2 hours before 92 mL of Milli-Q water was slowly added. The solution was then mixed with 280 mL of Milli-Q water and 8 mL of H_2_O_2_ (30%) until the color turned yellow. The supernatant was decanted, and the remaining solution was centrifuged at 3,000 rpm for 1 hour. The solid phase was rinsed with 4 M HCl solution (200 mL) for the final purification of graphite oxide. Additional treatment (i.e., sonification in Milli-Q water for 1 hour) was applied to obtain bare GO.

### 2.2. Magnetic GO Synthesis

Fe_3_O_4_ nanoparticles were prepared following a procedure previously reported in Wei and Wang [17]. In this study, magnetic GO was synthesized by mixing 0.05 g of Fe_3_O_4_ nanoparticles with the prepared graphite oxide having several stacked layers at a mass ratio of 9 : 1 in Milli-Q water followed by ultrasonication of the mixture solution for 20 minutes, where Fe_3_O_4_ nanoparticles are tightly attached to GO via nonspecific interaction rather than chemical pathways. Detailed procedure will be reported elsewhere. The solution was finally freeze-dried, and the remaining solids were used as a magnetic GO for the subsequent adsorption experiments. The synthesis of magnetic GO was confirmed by scanning electron microscopy (SEM, HITACHI S-4700), transmission electron microscopy (TEM, 200 kV FE-TEM-JEOL), X-ray diffraction (XRD, D/MAX-2500/PC, Rigaku), Raman spectroscopy (Renishaw 633 nm), and Fourier transform infrared spectroscopy (FT-IR, Perkin-Elmer spectrum 100).

### 2.3. Adsorption Experiments

Multiwalled CNT (MWCNT) and powdered activated carbon (PAC) were purchased from a supplier (Sigma-Aldrich) as the comparative adsorbents for our magnetic GO. The points of zero charge (pH_PZC_) values of all the adsorbents were estimated following an acid-base titration method suggested by Li et al. [3].

Three divalent metals Cu(II), Pb(II), and Cd(II) were used for the adsorbates. The three metals were all prepared in nitrate stock solutions at 1000 mg/L. Suwannee River fulvic acid (SRFA), which is a representative aquatic humic substance, was obtained from the International Humic Substance Society to examine influence of fulvic acids (10 mg/L) on the metal adsorption.

Batch adsorption experiments were conducted in triplicate by mixing each metal in a range of concentrations into 0.01 NaNO_3_ solution containing 0.4 g L^−1^ of the adsorbents (i.e., magnetic GO, MWCNT, and PAC). Adsorption was done by mixing the suspension solutions in a shaker at 150 rpm for 24 hours at a room temperature of 20 ± 1°C. The mixing time was chosen as an apparent equilibrium time for this study based on previous reports using GO [10, 18]. Different pH values were achieved to examine the solution pH effects on adsorption behaviors by adding 0.1 N HCl or 0.1 N NaOH solution with the maximum added volume maintained below 1% of the total volume. Competitive adsorption was evaluated by comparing the adsorption results for a single metal versus a mixture of three metals. After equilibrium, the supernatants were separated by filtering the solution through the 0.22 *μ*m membrane filter (cellulose acetate) prewashed with the samples. The concentrations of the metals in the filtrates were determined on ICP-OES (iCAP6300 Duo, Thermo, UK). Dissolved organic carbon (DOC) concentrations of SRFA were measured using a Shimadzu V-CPH analyzer. The adsorption amounts of either metal or SRFA on the adsorbents were calculated by the difference between the initial added concentrations and the corresponding residual amount after adsorption.

The adsorption data were fitted by the Langmuir (1) and Freundlich (2) models, and the related isotherm parameters were estimated:

(1) $$\begin{array}{c} q_{e}=\frac{q_{\max}\cdot k_{L}\cdot C_{e}}{1+k_{L}\cdot C_{e}}, \end{array}$$

(2) $$\begin{array}{c} q_{e}=k_{F}\cdot C_{e}^{1/n}, \end{array}$$

where *q*_*e*_ (mg g^−1^) and *q*_max⁡_ (mg g^−1^) are the equilibrium adsorption amount and the maximum adsorption capacity, respectively, and *C*_*e*_ (mg L^−1^) is the equilibrium metal concentration in solution. *k*_*L*_ (L mg^−1^) is the adsorption affinity related to adsorption energy; *k*_*F*_ and 1/*n* are the Freundlich model capacity factor and the Freundlich model site heterogeneity factor, an indicator of isotherm nonlinearity, respectively.

## 3. Results and Discussion

### 3.1. Characterization of Magnetic GO

**Figure 1:**
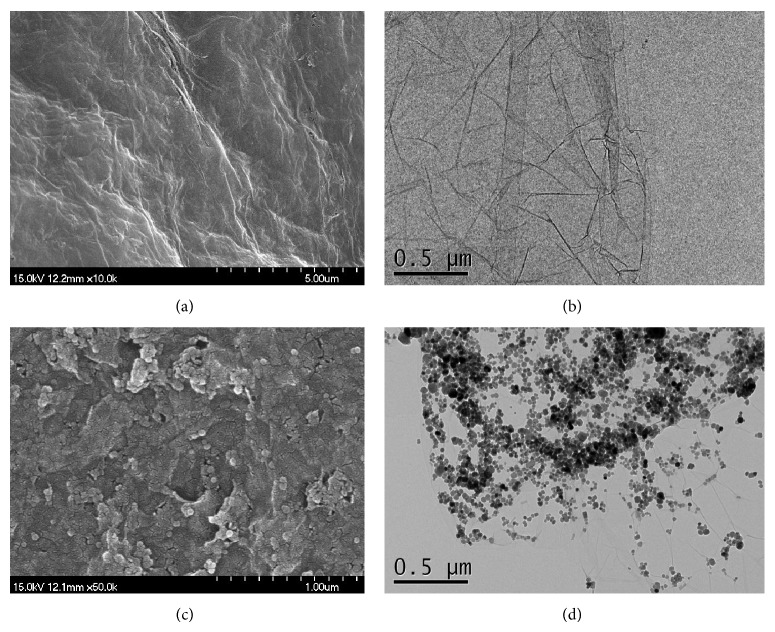
SEM (a) and TEM (b) images of the bare GO for this study. SEM (c) and TEM (d) images of the magnetic GO synthesized in this study.

**Figure 2:**
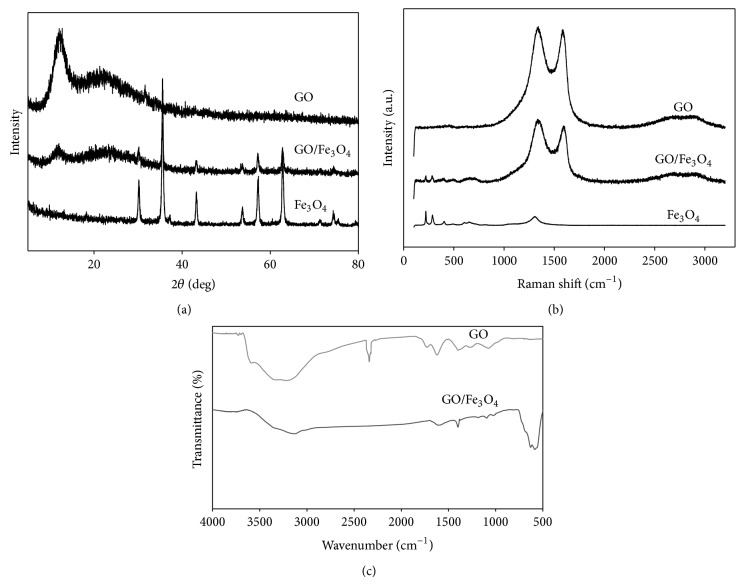
The XRD patterns (a), the Raman spectra (b), and the FT-IR spectra (c) of bare GO, the magnetic GO (GO/Fe_3_O_4_), and Fe_3_O_4_.

Comparison of the SEM and the TEM images between the bare GO and the magnetic GO revealed that Fe_3_O_4_ nanoparticles were successfully attached to the GO surfaces without the damage of the layered structures during the synthesis (Figure 1). The XRD patterns of GO, Fe_3_O_4_ nanoparticles, and the magnetic GO are displayed in Figure 2(a). The diffraction peaks of the GO/Fe_3_O_4_ composite are characterized by a combination of the individual peak patterns of GO and Fe_3_O_4_. For example, a unique peak of the bare GO appearing at 2*θ* = 12.1° was also shown for the peak pattern of the magnetic GO, and the other peaks of the magnetic GO appearing at 2*θ* = 30.2°, 35.5°, 43.2°, 53.7°, and 62.8° corresponded to those of the Fe_3_O_4_ nanoparticles. Our XRD results also indicate the successful composition of GO and Fe_3_O_4_, consistent with previous reports [5, 10, 19], in which ordered graphitic crystal structures of GO still remained after the functionalized processes with Fe_3_O_4_ nanoparticles. The Raman spectrum of the bare GO is characterized by two well-defined peaks at 1321 cm^−1^ and 1604 cm^−1^, each of which corresponds to disordered (D band) and ordered (G band) crystal structures of carbon [5]. The same peaks were found for our magnetic GO with the peaks of Fe_3_O_4_ partly superimposed (Figure 2(b)). The FT-IR spectra of the bare GO and the magnetic GO also confirmed the successful composition (Figure 2(c)). The spectrum of the magnetic GO consists of a unique peak of Fe_3_O_4_ at 570 cm^−1^, corresponding to the stretching vibration of Fe–O, and several weakened and/or split peaks of the bare GO, which include a broad peak at 3328 cm^−1^ (stretching of O–H), 1605 cm^−1^ (stretching of C=C), 1405 cm^−1^ (shifted stretching of C=O), and 1120 cm^−1^ (stretching of C–OH and the deformation of the C–O band) [5].

Based on acid-base titration curves (see Figure S1 in Supplementary Material available online at http://dx.doi.org/10.1155/2015/836287), the pH_PZC_ values of the adsorbents were estimated to be 4.5, 5.0, and 6.6 for the magnetic GO, MWCNT, and PAC, respectively. The pH_ZPC_ value of the magnetic GO corresponds to a middle point between the reported values of the bare GO and magnetite (i.e., Fe_3_O_4_) [20, 21]. The BET surface area of the magnetic GO was 49.9 m^2^/g, while those of MWCNT and PAC are reported to be 220 m^2^/g (from supplier) and 886 m^2^/g [22], respectively.

### 3.2. Influence of pH on Metal Removal by Adsorption

**Figure 3:**
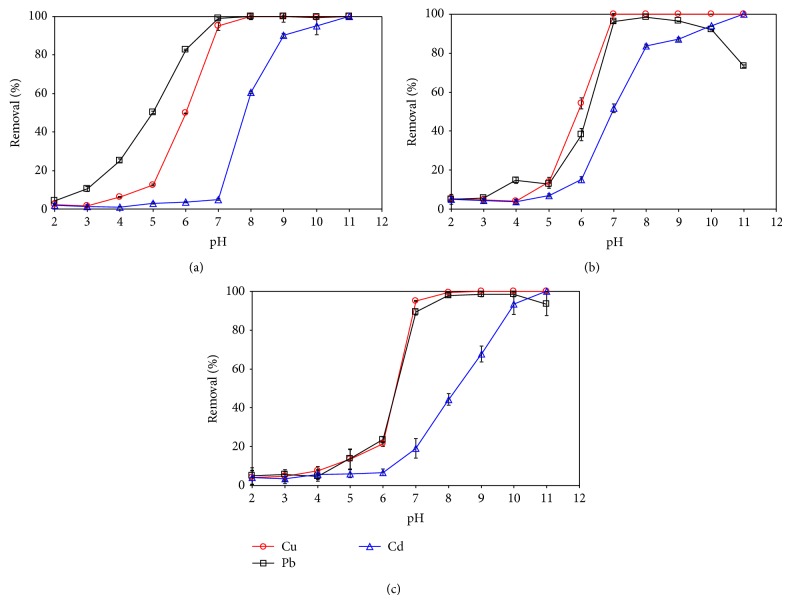
Effects of solution pH on the percent removals of three metals by adsorption upon magnetic GO (a), MWCNT (b), and PAC (c).

The effects of solution pH on the percent removal were compared for the types of the metals and the adsorbents in Figure 3. For all cases, the percent removal increased with a higher pH, and a sharp increase was found at the pH ranges around the pH_PZC_ value of each adsorbent. At pH < pH_ZPC_, the surfaces of the adsorbents are net positively charged due to the protonation of the acidic functional groups attached to carbon structures. The low adsorption levels at the lower pH ranges can be thus attributed to electrostatic repulsion between the net positively charged surfaces and the predominant metal species with a positive charge (i.e., Cu^2+^, Cd^2+^, and Pb^2+^) (Figure S2) and also to stronger competition between H^+^ and the metal ions for the available adsorption sites [23, 24]. At pH > pH_ZPC_, in contrast, the deprotonated sites on the surfaces become expanded with a higher pH, resulting in stronger attraction for positively charged metal ions present in the solution. The high level of adsorption at the higher pH ranges can also be driven by the precipitation of insoluble metal species on the surfaces and by surface ligand exchange of negatively charged metal species [3].

Despite the similar trends with pH, the individual responses of each metal adsorption to solution pH were different depending on the types of the metals as well as the adsorbents (Figure 3). For example, a higher removal was accomplished by adsorption in the order of Pb(II) > Cu(II) > Cd(II) for the magnetic GO at the pH around pH_PZC_, while the higher adsorption level of Pb(II) versus Cu(II) was not found for the other two adsorbents in the similar pH range (Figure 3). In addition, it was observed that the relative differences in the percent removal between the three metals were not the same for the different adsorbents at a particular pH. The slight decrease in adsorption of Pb(II) on MWCNT and PAC at very high pH ranges is possibly explained by the formation of negatively charged hydroxide complexes such as Pb(OH)_3_^−^ (Figure S2).

### 3.3. Adsorption Isotherms on Magnetic GO for Single-Metal Systems

**Figure 4:**
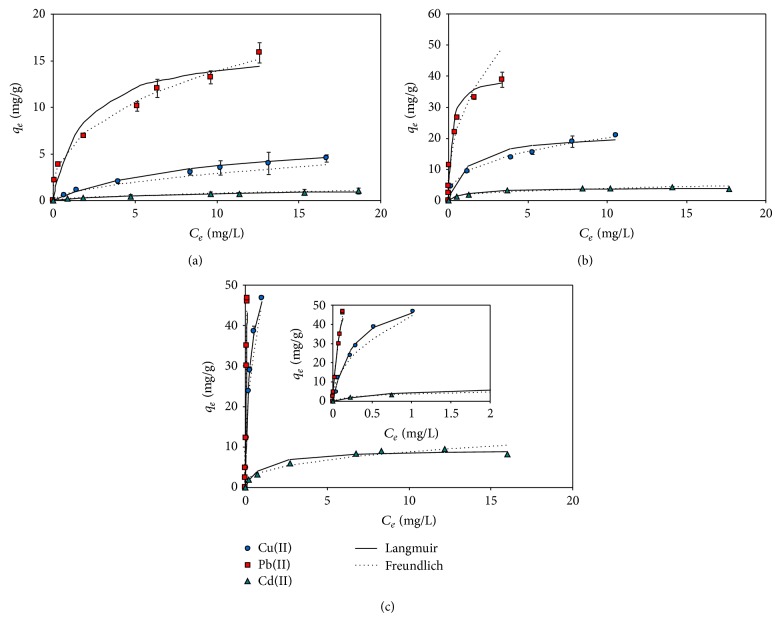
Adsorption isotherms of Cu(II), Pb(II), and Cd(II) on magnetic GO at pH 4.0 (a), 6.0 (b), and 7.0 (c) under the single-metal systems.

Equilibrium adsorption isotherms on the magnetic GO and the associated model parameters were compared for the three metals at different pH (4.0, 6.0, and 7.0) (Figure 4 and Table 1). Mostly, the Langmuir isotherm model fitted better the experimental data than the Freundlich model as revealed by the higher *R* ^2^ values (*R* ^2^ = 0.963 to 0.999), suggesting that the metal adsorption could be described by a monolayer adsorption process [9]. For all the pH conditions, the *q*_max⁡_ values of the magnetic GO were higher for the metals in the order of Pb(II) > Cu(II) > Cd(II), which were 15.48 ± 0.95, 6.24 ± 0.21, and 1.28 ± 0.03 mg g^−1^ at pH 4.0, 38.50 ± 3.16, 23.09 ± 1.45, and 4.41 ± 0.19 mg/g at pH 6.0, and 81.49 ± 9.94, 59.44 ± 3.64, and 9.92 ± 0.55 mg g^−1^ at pH 7.0 (Table 1). The relative order of the *q*_max⁡_ values agreed well with previous adsorption studies using MWCNT [23], peat [25], and magnetic GO [9]. Again, the increased metal adsorption at a higher pH can be attributed to the weakened competition between H^+^ and the positively charged metals for the adsorption sites (i.e., acidic functional groups) as well as to the decrease in the positive surface charge resulting in a lower degree of the electrostatic repulsion between the metals and the adsorbent. The *q*_max⁡_ values of this study were compared with those recently reported with magnetic GO (Table 2). The values were comparable to the range previously reported.

It is noteworthy that the order of the *q*_max⁡_ values is the same as that of the electronegativity of the metals, which are 2.33, 1.90, and 1.69 for Pb(II), Cu(II), and Cd(II), respectively [26]. This result strongly suggests that ion exchange may be a dominant mechanism to explain the metal adsorption. Diffusion may also operate as an important factor for the adsorption, controlling the transfer of metals to the adsorbent's surface [27]. In this case, smaller sized metals can easily penetrate into the boundary layers and/or the pores of the adsorbents, subsequently occupying more available sites. In this study, the adsorption levels of the metals were consistent with the decreasing order of the radii of the hydrated metals (Pb(II): 4.01 Å, Cu(II): 4.19 Å, and Cd(II): 4.26 Å), but not with that of the ionic radius, which correspond to 1.33 Å, 0.72 Å, and 0.97 Å for Pb(II), Cu(II), and Cd(II), respectively. Therefore, the metals surrounded by water molecules (i.e., hydrated form), not in the isolated forms, appear to participate in the actual adsorption processes. Overall, our explanation is supported by Baker [27], who suggested ion exchange and physical movement through diffusion as the major mechanisms for the adsorption of nickel(II) and copper(II) on silicate minerals.

The relative difference (or the relative ratio) of the *q*_max⁡_ values between Pb(II) and Cu(II) was greater at a lower pH, at which negatively charged surface sites are more depleted and the competition between the metals and hydrogen ions (and/or sodium ions) becomes greater for available adsorption sites. Pb(II) exhibited the highest adsorption affinity (i.e., *K*_*L*_) among the three metals, although Cu(II) and Cd(II) were not discriminated in the *K*_*L*_ values at pH 4.0 and 6.0.

### 3.4. Competitive Adsorption Isotherms on Magnetic GO at Different pH

**Figure 5:**
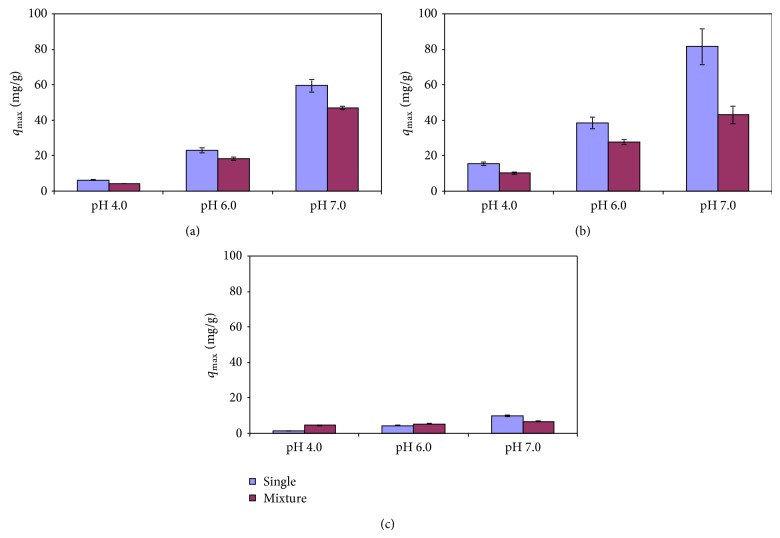
Comparison of the maximum adsorption capacities of Cu(II), Pb(II), and Cd(II) on magnetic GO between the single- and multimetal adsorption at pH 4.0 (a), 6.0 (b), and 7.0 (c).

The Langmuir isotherm model was applied to the adsorption onto the magnetic GO for multimetal systems containing the three metals, and the calculated isotherm parameters were compared with those of the single-metal systems (Table 1; Figure 5). Similar approaches have been frequently used in many prior studies to evaluate the competition adsorption of metals [18, 28–31].

In this study, the total adsorption was generally higher for the multimetal systems than for the single-metal systems, while the adsorption of the individual metal was diminished when the three metals were present together. Among the three metals, the greatest decrease (or antagonistic effect) was found for Pb(II) adsorption at pH 7.0 (~47% reduction). One exception was Cd(II) adsorption at pH 4.0 and 6.0, showing enhanced adsorption (Figure 5). The decreased adsorption suggests that there is competition between the three metals for the same adsorption sites. Our results agreed with other previous studies reporting the antagonistic effects on multimetal adsorption [23, 25, 31]. In our multimetal systems, Pb(II) still had the highest maximum adsorption capacity on the magnetic GO at pH 4.0 and 6.0. At pH 7.0, however, Cu(II) exhibited a slightly higher adsorption level than Pb(II). These results indicate that the preference of the magnetic GO surfaces for metals may depend on the solution pH, probably resulting from variable surface characteristics with pH. The *q*_max⁡_ values of Pb(II) and Cu(II) were lower for the multi- versus single-metal systems independent of the pH, while Cd(II) adsorption was dependent on the pH. For example, when the adsorption changed from the single-metal to the multimetal systems at pH 6.0, the maximum adsorption levels dropped by ~20% from 23.1 ± 1.5 to 18.4 ± 1.1 mg g^−1^ for Cu(II) and by ~28% from 38.5 ± 3.2 to 27.7 ± 1.2 mg g^−1^ for Pb(II). For Cd(II), however, they increased by ~21% from 4.4 ± 0.2 to 5.3 ± 0.2 mg g^−1^ (Table 1). The enhanced adsorption capacity of Cd(II) was found at pH 4.0 and 6.0 but not at pH 7.0. Therefore, there seems to be no consistent trend for the competitive adsorption with either pH or the type of the metals for this study.

Our observation of the competitive effects did not agree with those expected from the relative adsorption affinity of each metal that follows in the order of Pb(II) > Cu(II) ~ Cd(II), assuming that a metal with a higher adsorption affinity would displace others with a lower affinity on adsorbents when the metals compete for the same adsorption sites [18, 31]. Therefore, some other factors than adsorption affinity may be responsible for the competitive adsorption behaviors. In fact, there is no consensus concerning competitive adsorption of metals [26]. Previous literature suggests that antagonistic effects on multimetal adsorption could depend on the type of metals and adsorbents. Sitko et al. [18] have demonstrated that the adsorption rate of Pb(II) onto GO was the lowest among the metals including Cu(II) and Cd(II). This suggests that the low adsorption rate of Pb(II) may be responsible for the greatest antagonistic effects observed for Pb(II). Further investigations are warranted to provide concrete evidence.

### 3.5. Competitive Adsorption of Metals on Different Adsorbents

Competitive adsorption isotherms of the three metals were compared for three different adsorbents at pH 7.0 by examining their Langmuir model parameters (Table 3; Figure S4). Similarly to the magnetic GO, although the total adsorption of the three metals was higher for the multimetal systems, the individual metal adsorption was lower on either PAC or MWCNT compared to the single-metal systems. The degree of the reduction in the *q*_max⁡_ values was in the sequence of the magnetic GO > MWCNT > PAC for Cu(II) and Pb(II), which have relatively high electronegativity and small hydrated radius. The maximum adsorption levels of Cu(II) decreased by approximately 21%, 12%, and 6% for the magnetic GO, MWCNT, and PAC, respectively, while, for Pb(II), they decreased by ~47%, ~18%, and ~0.2%. The different competitive adsorption is probably due to the differences in the distributions of available adsorption sites on the surfaces for each metal. Plazinski and Rudzinski [32] have adopted several mathematical isotherm models to demonstrate the effects of surface heterogeneity on the adsorption of heavy metals based on a premise that the surface sites are not energetically and chemically uniform. In this context, the magnetic GO surfaces exhibiting the highest reduction for the multimetal systems are likely to be the most uniform with respect to the active sites for Cu(II) and Pb(II). In contrast, the adsorption sites of PAC can be considered to be more heterogeneously distributed with the least pronounced antagonistic effects. More accessible sites for metals in small pores of PAC could contribute to the least antagonistic effects on the competitive adsorption among the three adsorbents [33]. Meanwhile, the same trend with the adsorbents was not found for Cd(II), in which 32%, 23%, and 39% decreases were shown for the magnetic GO, MWCNT, and PAC, respectively. The different competitive adsorption of Cd(II) could relate to such properties of the metal as the largest hydrated radius and the lowest electronegativity, which needs further investigations.

### 3.6. Effects of Fulvic Acid on Metal Adsorption

Effects of the presence of aquatic fulvic acid on the metal adsorption were examined for the different adsorbents (Table S1). DOC measurements of the residual SRFA in solution after adsorption indicate that SRFA was adsorbed by approximately 90%, 80%, and 50% onto the magnetic GO, PAC, and MWCNT, respectively, for this study under a particular environmental condition. The presence of humic substances (HS) can affect metal adsorption behavior by modifying the surface characteristics of adsorbents and/or by forming complexes with dissolved metals in solution. Previous studies have shown that the adsorption of metals onto adsorbents can be enhanced by the presence of HS at relatively low pH mainly due to strong complexation between the negatively charged HS adsorbed on surfaces and the metals in solution [34]. This effect tends to be more evident for the pH range lower than the pH_ZPC_ of adsorbents. Meanwhile, dissolved HS also play roles in the metal adsorption, competing with active surface sites for complexation with metals. This competition effect is likely to be more pronounced for the pH range higher than the pH_ZPC_ of adsorbents, at which the surfaces of adsorbents are net negatively charged similarly to HS [3, 34]. Because the concentration of the residual SRFA after adsorption was higher in the order of MWCNT > PAC > magnetic GO for this study, the competition effects of the residual SRFA on metal complexation with surfaces, if any, would be greater in the same order. For a detailed investigation, an evaluation index, *α*, was introduced to qualitatively track the SRFA-induced changes in the adsorption affinity of metals as a function of the initial metal concentrations. Here, *α* was calculated based on the following equation:

(3) $$\begin{array}{c} \alpha =\frac{q_{e,\text{SRFA}}/C_{e,\text{SRFA}}}{q_{e}/C_{e}}, \end{array}$$

where *α* is the evaluation index for SRFA effects on metal adsorption affinity; *q*_*e*,SRFA_ and *q*_*e*_ are the equilibrium adsorption capacities in the presence and the absence of SRFA, respectively. *C*_*e*,SRFA_ and *C*_*e*_ are the equilibrium concentrations of metals in solution.

**Figure 6:**
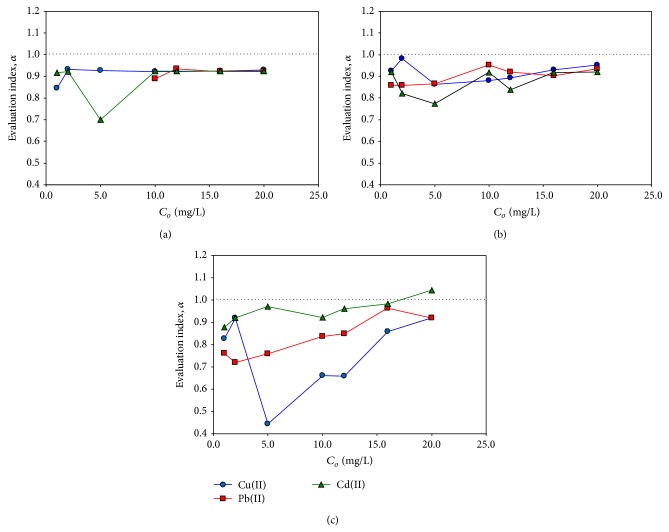
The evaluation index (a) for SRFA effects on metal adsorption on magnetic GO (a), PAC (b), and MWCNT (c) as a function of the initial metal concentrations (*C*_*o*_). The first three data points of Pb(II) on the magnetic GO were neglected because of the extremely low equilibrium concentrations (*C*_*e*_).

The evaluation index was plotted against the initial metal concentrations in Figure 6. The index was mostly lower than the unity, varying from 0.70 to 0.93 for Cu(II) with the mean value of 0.87 ± 0.13, from 0.77 to 0.95 for Pb(II) with 0.88 ± 0.07, and from 0.66 to 1.04 for Cd(II) with 0.91 ± 0.09. These results indicate that the presence of SRFA lowered the metal adsorption on all the adsorbents, and they suggest that the residual SRFA had greater effects on the overall metal adsorption than the adsorbed SRFA. The lowest value (0.76 ± 0.17) of the index was found for Cu(II) adsorption onto MWCNT, which was statistically distinguished from the metal adsorption onto the other two adsorbents (ANOVA *P* < 0.05), probably resulting from the high concentration of the residual SRFA. Such a significant difference in the index among the three adsorbents, however, was not found for the other two metals (ANOVA *P* > 0.05). This result may be attributed to the much higher complexation capability of SRFA for Cu(II) compared to Pb(II) and Cd(II) [35]. Our results demonstrate that the effects of HS on metal adsorption could depend on the HS concentration in aqueous phase, which varies with solution pH and the adsorbents, as well as on the type of the metal.

## 4. Conclusions

Magnetic GO was successfully synthesized by a simple ultrasonification method using graphite oxide and Fe_3_O_4_ nanoparticles. The adsorption of Cu(II), Pb(II), and Cd(II) all increased with a higher pH, with sharp changes at the pH values around the pH_ZPC_ of each adsorbent. The equilibrium adsorption was better described by the Langmuir isotherm model than the Freundlich model, indicating that monolayer adsorption processes can explain the metal adsorption. The *q*_max⁡_ values of the metals on the magnetic GO were higher in the order of Pb(II) > Cu(II) > Cd(II). The relative order of the *q*_max⁡_ values was consistent with the sequence of the electronegativity and the hydrated radius of the metals. The adsorption of the individual metal was mostly reduced when other metals coexisted, indicating that the competition among the metals exists for the adsorption sites. However, the trend of the reduction with solution pH and the degree were different for each metal. Comparison of the *q*_max⁡_ values between the single- and multimetal systems of Cu(II) and Pb(II) revealed that the competitive adsorption was greater in the order of the magnetic GO > MWCNT > PAC. For all the three adsorbents, the presence of SRFA decreased the adsorption affinities of the metals, suggesting that dissolved HS play a greater role than adsorbed HS in metal adsorption by competing with active surface sites for the complexation with metals.

## Supplementary Material

The Supplementary Materials contains 4 figures and 1 table, which are the figures of acid-base titration curves for the three different adsorbents (i.e., magnetic GO, MWCNT, and PAC), the figures showing the percent distributions of soluble metal species at different pH, the figures of adsorption isotherms of Cu(II), Pb(II), and Cd(II) on magnetic GO at different pH under multimetal system, the figures comparing the maximum adsorption capacities of different adsorbents for the metals, and a table displaying isotherm model parameters of metal adsorption in the presence of SRFA.

## Figures and Tables

**Table 1:** Isotherm model parameters of the metal adsorption on the magnetic GO.

System	Metals	pH	Langmuir			Freundlich		
			*q*_max⁡_^a^	*k*_*L*_^b^	*R* ^2^	*k*_*F*_^c^	1/*n* ^d^	*R* ^2^
Single metal	Cu(II)	4.0	6.24 ± 0.21^e^	0.13 ± 0.01^e^	0.998	0.96 ± 0.08^e^	0.55 ± 0.03^e^	0.992
	Pb(II)		15.48 ± 0.95	0.63 ± 0.17	0.981	5.91 ± 0.29	0.37 ± 0.02	0.991
	Cd(II)		1.28 ± 0.03	0.14 ± 0.01	0.999	0.21 ± 0.02	0.52 ± 0.04	0.985
	Cu(II)	6.0	23.09 ± 1.45	0.59 ± 0.14	0.987	8.59 ± 0.42	0.39 ± 0.03	0.993
	Pb(II)		38.50 ± 3.16	4.62 ± 1.72	0.963	27.97 ± 1.28	0.31 ± 0.04	0.967
	Cd(II)		4.41 ± 0.19	0.64 ± 0.12	0.981	1.89 ± 0.29	0.29 ± 0.07	0.856
	Cu(II)	7.0	59.44 ± 3.64	3.47 ± 0.54	0.991	48.43 ± 1.91	0.46 ± 0.04	0.982
	Pb(II)		81.49 ± 9.94	8.41 ± 1.97	0.992	151.17 ± 31.26	0.63 ± 0.09	0.96
	Cd(II)		9.92 ± 0.55	0.69 ± 0.18	0.974	4.18 ± 0.59	0.31 ± 0.06	0.899

Multimetal	Cu(II)	4.0	4.33 ± 0.08	0.16 ± 0.01	0.999	0.77 ± 0.10	0.53 ± 0.05	0.977
	Pb(II)		10.27 ± 0.72	0.73 ± 0.25	0.967	4.41 ± 0.39	0.32 ± 0.04	0.964
	Cd(II)		4.59 ± 0.15	0.11 ± 0.01	0.999	0.58 ± 0.07	0.60 ± 0.05	0.984
	Cu(II)	6.0	18.37 ± 1.07	1.36 ± 0.44	0.975	9.36 ± 0.87	0.30 ± 0.05	0.945
	Pb(II)		27.71 ± 1.19	7.05 ± 2.00	0.980	20.45 ± 1.18	0.22 ± 0.04	0.942
	Cd(II)		5.34 ± 0.21	0.24 ± 0.03	0.996	1.26 ± 0.08	0.47 ± 0.03	0.992
	Cu(II)	7.0	46.77 ± 0.96	4.04 ± 0.26	0.998	36.13 ± 2.07	0.39 ± 0.06	0.951
	Pb(II)		43.03 ± 4.83	64.84 ± 33.86	0.911	50.63 ± 2.07	0.17 ± 0.02	0.981
	Cd(II)		6.78 ± 0.32	0.98 ± 0.24	0.976	3.12 ± 0.21	0.31 ± 0.03	0.975

^a^Maximum adsorption capacity estimated by Langmuir isotherm model (mg g^−1^).

^b^Adsorption affinity estimated by Langmuir isotherm model (L mg^−1^).

^c^Freundlich model capacity ((mg/g) (L/mg)^1/*n*^).

^d^Freundlich model site heterogeneity factor (dimensionless).

^e^Standard errors.

**Table 2:** Comparison of Cu(II), Pb(II), and Cd(II) adsorption capacities of this study with other recent reports using magnetic GO.

Sorbents	Environmental conditions	Metals	*Q*_max⁡_ (mg/g)	References
GO/Fe_3_O_4_	pH = 5.3, *T* = 20°C	Cu(II)	18.3	Li et al. [3]

GO/silica/Fe_3_O_4_	pH = 7.1, *T* = 25°C	Pb(II)	333.3	Wang et al. [9]
		Cd(II)	166.7	

GO/Fe_3_O_4_/sulfanilic acid	pH =6.0, *T* = 25°C	Cd(II)	55.4	Hu et al. [7]

GO-MnFe_2_O_4_	pH =5.0, *T* = 25°C	Pb(II)	673	Kumar et al. [11]

Reduced GO/CoFe_2_O_4_	pH = 5.3, *T* = 25°C	Pb(II)	299.4	Zhang et al. [12]
	pH = 5.3, *T* = 35°C		274.7	
	pH = 5.3, *T* = 45°C		253.2	

GO/Mn-doped Fe(III) oxide	pH = 5.0, *T* = 25°C	Cd(II)	87.2	Nandi et al. [8]
		Cu(II)	129.7	

GO/Fe_3_O_4_/sulfanilic acid	pH = 5.0, *T* = 20°C	Cu(II)	50.7	Hu et al. [6]
	pH = 5.0, *T* = 30°C		56.9	

GO/Fe_3_O_4_	pH = 6.0, *T* = 20°C	Cu(II)	23.1	This study
		Pb(II)	38.5	
		Cd(II)	4.4	

**Table 3:** Isotherm model parameters of the metal adsorption on PAC and MWCNT at pH 7.0.

Adsorbents	Systems	Metals	Langmuir			Freundlich		
			*q*_max⁡_^a^	*k*_*L*_^b^	*R* ^2^	*k*_*F*_^c^	1/*n* ^d^	*R* ^2^
PAC	Single metal	Cu(II)	34.72 ± 1.47^e^	0.94 ± 0.13^e^	0.993	15.54 ± 1.54^e^	0.40 ± 0.07^e^	0.931
		Pb(II)	35.15 ± 1.15	1.30 ± 0.14	0.995	17.04 ± 1.60	0.39 ± 0.07	0.925
		Cd(II)	11.23 ± 0.38	0.11 ± 0.01	0.999	1.40 ± 0.11	0.60 ± 0.03	0.994
	Multimetal	Cu(II)	32.79 ± 0.76	0.73 ± 0.05	0.998	12.93 ± 0.99	0.44 ± 0.05	0.970
		Pb(II)	35.08 ± 0.38	0.617 ± 0.02	0.999	12.69 ± 0.98	0.46 ± 0.05	0.972
		Cd(II)	6.84 ± 0.20	0.10 ± 0.01	0.999	0.85 ± 0.09	0.595 ± 0.04	0.988

MWCNT	Single metal	Cu(II)	41.42 ± 0.58	0.72 ± 0.03	0.999	17.49 ± 0.98	0.41 ± 0.04	0.988
		Pb(II)	59.08 ± 0.37	1.22 ± 0.02	0.999	30.95 ± 0.96	0.57 ± 0.05	0.983
		Cd(II)	23.07 ± 0.97	0.13 ± 0.01	0.998	3.34 ± 0.45	0.60 ± 0.06	0.976
	Multimetal	Cu(II)	36.53 ± 2.63	1.13 ± 0.33	0.985	19.11 ± 0.54	0.32 ± 0.02	0.994
		Pb(II)	48.21 ± 2.00	0.36 ± 0.03	0.998	12.53 ± 0.57	0.58 ± 0.03	0.993
		Cd(II)	17.86 ± 0.56	0.11 ± 0.01	0.999	2.26 ± 0.27	0.62 ± 0.05	0.983

^a^Maximum adsorption capacity estimated by Langmuir isotherm model (mg g^−1^).

^b^Adsorption affinity estimated by Langmuir isotherm model (L mg^−1^).

^c^Freundlich model capacity ((mg/g) (L/mg)^1/*n*^).

^d^Freundlich model site heterogeneity factor (dimensionless).

^e^Standard errors.
