# Supplementary material for: Competitive Adsorption of Metals onto Magnetic Graphene Oxide: Comparison with Other Carbonaceous Adsorbents
Source: ScientificWorldJournal. 2015 Mar 12;2015:836287. doi: 10.1155/2015/836287 (PMC4377472; doi:10.1155/2015/836287)
Supplement: Supplementary file 1 — The Supplementary Materials contains 4 figures and 1 table, which are the figures of acid-base titration curves for the three different adsorbents (i.e., magnetic GO, MWCNT, and PAC), the figures showing the percent distributions of soluble metal species at different pH, the figures of adsorption isotherms of Cu(II), Pb(II), and Cd(II) on magnetic GO at different pH under multimetal system, the figures comparing the maximum adsorption capacities of different adsorbents for the metals, and a table displaying isotherm model parameters of metal adsorption in the presence of SRFA. [file 836287.f1.docx]

Supplementary Materials

*The Scientific World Journal*

Competitive Adsorption of Metals onto Magnetic Graphene Oxide: Comparison with Other Carbonaceous Adsorbents

Jin Hur^1,*^, Jaewon Shin^1^, Jeseung Yoo^2^, Young-Soo Seo^2^

*^1^Department of Environment & Energy, Sejong University, Seoul 143-747, South Korea*

*^2^Department of Nano materials, Sejong University, Seoul, 143-747, South Korea*

* Corresponding author: Tel. +82-2-3408-3826. Fax +82-2-3408-4320.

E-mail: jinhur@sejong.ac.kr

Figure S1. Acid-base titration curve for GO.

Fig. S1. Acid-base titration curves for magnetic GO (a), MWCNT(b), and PAC (c) for the determination of pH_ZPC_.

Fig. S2. Percent distributions of soluble metal species as a fucntion of solution pH at 20^o^C. (a) Cu, (b) Cd, and (c) Pb. The data were generated by using Visual MINTEQ (Vers. 3.0).

Fig. S3. Adsorption isotherms of Cu(II), Pb(II), and Cd(II) on magnetic GO at pH 4.0 (a), 6.0 (b), and 7.0 (c) for multi-metal systems.

Fig. S4. Comparison of the maximum adsorption capacities of different adsorbents for Cu(II), Pb(II), and Cd(II) between the single and the multi-metal adsorption at pH 7.0 (a) Magnetic GO, (b) PAC, and (c) MWCNT.

Table S1. Isotherm model parameters of metal adsorption in the presence of SRFA (10 mg C/L) for the multi-metal systems at pH 7.0.

| Adsorbents | Metals | Langmuir | | |  | Freundlich | | |
| --- | --- | --- | --- | --- | --- | --- | --- | --- |
|  |  | q_max_^a^ | k_L_^b^ | R^2^ |  | k_F_^c^ | 1/n^d^ | R^2^ |
| Magnetic GO | Cu(II) | 43.28±0.89^e^ | 4.04±0.01^e^ | 0.998 |  | 33.46±1.90^e^ | 0.40±0.06^e^ | 0.952 |
|  | Pb(II) | 39.84±4.48 | 0.01±0.01 | 0.911 |  | 46.88±1.92 | 0.17±0.02 | 0.981 |
|  | Cd(II) | 6.63±0.67 | 1.47±0.71 | 0.922 |  | 2.63±0.31 | 0.34±0.05 | 0.943 |
| PAC | Cu(II)  Pb(II)  Cd(II) | 31.54±1.22  33.36±0.97  7.24±0.77 | 1.61±0.18  1.80±0.15  13.44±2.61 | 0.996  0.998  0.994 |  | 11.56±0.62  11.40±1.01  0.67±0.09 | 0.47±0.04  0.48±0.06  0.65±0.05 | 0.987  0.967  0.986 |
|  | Cu(II) | 68.62±24.04 | 7.34±3.98 | 0.973 |  | 9.34±1.29 | 0.68±0.10 | 0.970 |
| MWCNT | Pb(II) | 57.11±6.07 | 4.80±0.88 | 0.995 |  | 10.11±0.68 | 0.67±0.05 | 0.989 |
|  | Cd(II) | 16.81±0.57 | 8.75±0.57 | 0.999 |  | 2.04±0.23 | 0.65±0.05 | 0.986 |

^a^ maximum adsorption capacity estimated by Langmuir isotherm model (mg/g)

^b^ adsorption affinity estimated by Langmuir isotherm model (L/mg)

^c^ Freundlich model capacity ((mg/g)(L/mg)^1/n^)

^d^ Freundlich model site heterogeneity factor (dimensionless)

^e^ Standard errors
